# How Do Perceived Changes in Child and Adolescent Activities Relate to Perceptions of Health during COVID-19? Exploring Heterogeneity during the Pandemic

**DOI:** 10.3390/ijerph191811206

**Published:** 2022-09-06

**Authors:** Amanda S. Gilbert, Jason Jabbari, Racquel Hernández

**Affiliations:** Social Policy Institute, Brown School, Washington University in St. Louis, St. Louis, MO 63130, USA

**Keywords:** COVID-19, screen time, extracurricular activities, school activities, physical health, mental health, children

## Abstract

COVID-19 affected child/adolescent activities (e.g., extra-curricular, screen time), along with physical health (PH) and mental health (MH); however, less is known about the relationship between changes in activities and PH and MH in the United States and how these relationships vary by race/ethnicity. To address this gap, data were used from a national survey (Socio-Economic Impacts of COVID-19 Survey) administered May–June 2021 (n = 853). Multinomial logistic regression explored changes in outdoor, school, extracurricular, friend, and screen time activities with changes in PH and MH; interactions explored moderation by race/ethnicity. Results showed increases in outdoor (RRR 2.36, *p* = 0.003), school (RRR 3.07, *p* < 0.001), and extracurricular activities (RRR 3.05, *p* < 0.001), which were associated with increases in chances of better PH. Better MH was more likely for children/adolescents where friend activities (RRR 3.34, *p* < 0.001) and extracurriculars (RRR 4.48, *p* < 0.001) increased. Except for extracurriculars, heterogeneous relationships were observed (e.g., increases and decreases in activities were simultaneously related to better and worse health). The relationship between outdoor activities and screen time with health were moderated by race/ethnicity. Findings support facilitating outdoor, school, extracurricular, and friend activities, which were positively related to health. Given heterogeneity and variation by race/ethnicity, more research is needed to understand the complex relationship between activities and health during COVID-19.

## 1. Introduction

The COVID-19 pandemic has disrupted the activities (e.g., school, extracurricular, screen time, time with friends) of many children and adolescents, as well as their physical health (PH) and mental health (MH). Research conducted during the pandemic found that activities such as screen time increased, while activities such as time spent outdoors, time spent with friends, time spent at school, and time spent participating in extracurricular activities decreased [1,2,3,4,5]. Additionally, children and adolescents’ PH and MH were found to decrease during the COVID-19 pandemic [6,7,8,9,10]. In one systematic review, it was estimated that child depression nearly doubled from 13% to 25% and anxiety from 12% to 21% [8]. Other studies have found obesity rates in children and adolescents increased from 19% to 22% within the first year of the pandemic, while physical activity and nutrition decreased [5,11,12].

In the United States, these disruptions to child and adolescent activities and changes in PH and MH have been inequitably distributed across race/ethnicity [6,13,14,15,16,17,18,19]. School closures and provision of online or in-person schooling varied by race/ethnicity, as did access to various school activities [12,13,20]. Prevalence of MH disorders (e.g., anxiety and depression) have also been found to be much higher among Black and Hispanic children and adolescents [21]. Research also indicates Black children and adolescents experienced greater increases in obesity, lower levels of physical activity, and worse nutrition during the COVID-19 pandemic [5,9,12,22].

Having a better understanding of these changes in child activities and PH and MH during the pandemic is important for three main reasons. First, changes in child and adolescent activities are associated with changes in child and adolescent PH and MH, potentially creating an unhealthy feedback loop between changes in activities and changes in health [1,23,24,25,26]. For example, research has shown that outdoor activities and taking part in extracurriculars (e.g., group sports) may increase physical activity and benefit child development, potentially improving PH and MH [27,28]. Additionally, spending time with friends is important for child and adolescent well-being [29]. Second, poor PH and MH in childhood can increase the risk of chronic disease later in life [30,31,32]. Third, the differential impacts of COVID-19 on child and adolescent activities and PH and MH across race/ethnicity could potentially lead to widening health disparities [16,20]. 

While research has been conducted on child and adolescent activities and health during COVID-19, much of the focus has been either on PH or MH [33,34], and the research conducted on both PH and MH is largely outside the United States [35,36]. As such, less is known about the relationship between *changes* in child and adolescent activities and PH and MH in the United States, as well as the role of race/ethnicity in these relationships. To fill this gap, this study aims to (1) describe changes in child and adolescent activities and changes in PH and MH during COVID-19 for children aged 5–18 by race/ethnicity; (2) assess the relationship between changes in child and adolescent activities with changes in both PH and MH; and (3) explore how these relationships are moderated by race/ethnicity. We hypothesize that decreases in outdoor, school, extracurricular, and friend activities and increases in screen time during the COVID-19 pandemic will be associated with worse PH and MH, and that there will be differences in these relationships across race/ethnicity. 

## 2. Materials and Methods

### 2.1. Study Design 

The study was conducted from May 2021 through June 2021. During this time, while there was variability in COVID-19 incidence across the United States, cases were declining from the wave in January of 2021 [37]. This decline occurred in large part due to the availability and distribution of vaccines in late winter and early spring of 2021 [38]. Across the United States, as vaccine coverage increased, COVID-19 restrictions lifted. By late June 2021, only eleven states had mask mandates, four states had gathering bans, and none had mandated stay-at-home orders [39]. 

We conducted a cross-sectional analysis using the final wave (12 May 2021–21 June 2021) of the Socio-Economic Impacts of COVID-19 Survey. The previous four waves of data collection occurred from 22 April to 2 June 2020 (Wave 1), 30 July to 9 September 2020 (Wave 2), 3 November to 29 December 2020 (Wave 3), and 4 February through 18 March 2021 (wave 4). Survey questions were designed to elicit information on household characteristics and on parent perceptions regarding changes in their children’s social and emotional wellbeing since the start of the pandemic. For simplicity, and since parents were reporting on their children, we refer to children and adolescents as children throughout the rest of the paper. Participants were recruited using quota sampling methods from a large, online panel provider, so that the overall survey sample would be nationally representative in terms of gender, race/ethnicity, income, and age. Participants were invited into the survey via email, and consent was obtained at the start of the survey, which was completed online and lasted roughly 20 minutes. Parents with multiple children were randomly assigned to respond for a specific child. Upon completion of the survey, participants were provided incentives. Participants were required to be at least 18 years or older and live in the United States. This study received full approval of the university’s Institutional Review Board.

The survey sample consisted of 5054 participants. We excluded participants who did not have children (n = 3512) or whose children were too young or too old for school (n = 327), creating a sample of 1215. We excluded children who were too young or too old for school because some activities were school-related (e.g., spending time on school/homework) and because we wanted the sample to consist of school-aged children. After a listwise deletion for missing observations (n = 362), our final analytic sample consisted of 853 participants.

### 2.2. Measures

#### 2.2.1. Changes in MH and PH 

Parents were asked how their child’s PH and MH had changed since the start of the COVID-19 pandemic, with response options of 1 = Much worse through 5 = Much better. Due to sample size and for the purposes of providing more easily interpretable results, each of these variables was collapsed into three categories 0 = Worse (much worse, somewhat worse), 1 = No change (stayed the same), 2 = Better (somewhat better, much better).

#### 2.2.2. Changes in Child Activities 

To assess changes in activities we asked parents “How has the way your child spends their time on each of the following activities changed since the start of the COVID-19 pandemic?” Activities included spending time outdoors; spending time on computers, smartphones, or other electronic devices; spending time on school/homework; spending time with friends; and spending time on extracurricular activities (e.g., sports, music). Response options ranged from 1 = Large decrease through 5 = Large increase. For analyses, each of these variables was collapsed into three categories due to sample size and for ease of interpretation: 0 = Decrease (large decrease, moderate decrease), 1 = No change (no change), 2 = Increase (moderate increase, large increase). 

#### 2.2.3. Covariates

Covariates included participant *gender* (Male, Female), *child grade* (Kindergarten, 1st through 2nd grade, 3rd through 5th grade, 6th through 8th grade, and 9th through 12th grade), *primary language* (English, Spanish/other), *number of children* (one, More than one), *employment* (full time, part time, not working), *spouse employment* (single, full time, part time, not working), *school plan* (in-person only, online only, Mix of in-person and online, choice of in-person and online), *school quality before and during COVID-19* (poor, average, good), *COVID-19 incidence* (cumulative number of cases per county, logged), and *population density by county* (thousands per sq. mile). We also asked about the highest education level of the parent or their partner to capture educational attainment termed *bachelor’s degree* (less than a bachelor’s degree, bachelor’s degree or higher). *Income* was measured using household’s total income in 2020, household size, and the US Department of Housing and Urban Development (HUD)’s measure of area median income (AMI) at the county level [40]. Income levels included (0–50% of AMI, 50–80% of AMI, 80–120% of AMI, 120%+ of AMI). *Job loss* was coded as (Yes, No), with yes coded if either or both parents reported job loss due to COVID-19. To measure race, participants were able to choose multiple options (White/Caucasian, Black/African American, Asian, Native American/Pacific Islander, or some other race). Participants were separately asked if they were Hispanic or Latino/a/x (Yes, No), to assess ethnicity. If yes, participants were coded as such regardless of responses to racial categories. Due to small sample size of Native American/Pacific Islander and some other races, *race/ethnicity* for this analysis included White/Caucasian, Black/African American, Hispanic, Asian.

### 2.3. Analysis 

We ran descriptive statistics using frequencies and chi-squared tests to describe changes in child activities and PH and MH, and to determine if there were significant associations between changes in activities with changes in PH and MH. Next, we assessed associations between changes in activities with changes in PH and MH using two separate adjusted multinomial logistic regression models for each outcome (PH and MH). These models were adjusted for COVID-19 incidence, population density, parent and child demographics (respondent’s gender, child grade, primary language, number of children, employment, education, income, job loss, race/ethnicity), and school covariates (school plan, school quality) to account for variability in the outcomes of changes in PH and MH explained by these factors. We then ran the same adjusted multinomial logistic regressions using interaction terms to explore how the relationship between changes in child activity and child PH and MH might vary by race/ethnicity. 

## 3. Results

### 3.1. Descriptive Statistics 

Descriptive statistics are presented in Table 1. The analytic sample consisted of 853 participants. When considering the entire sample, as seen in column one, 58% of participants identified as White, 24% as Hispanic, 12% as Black, and 7% as Asian. Half of the sample (54%) identified as female. Most parents had a bachelor’s degree (78%), spoke English (95%), and had more than one child (54%); 18% were single. Regarding household finances, 25% of participants had earnings in 0–50% AMI range, and 34% experienced job or income loss. In terms of education, 27% of children were between pre-school and 2nd grade, 40% were between 3rd and 8th grade, and 33% were between 9th and 12th grade. Thirty-one percent of children were receiving online-only instruction, with most (60–66%) parents reporting school quality to be good before and during COVID-19. Around 48–68% of respondents reported having home resources associated with learning (online learning tools, adult learning assistance, broadband, a quiet place to study). 

As presented in column one of Table 1, 15% of children experienced worse PH and 30% experienced better PH. Fewer children (20%) experienced worse MH compared to 25% who experienced better MH. Regarding changes in child activities, a smaller percentage of children (29%, 17%, and 10%) experienced decreases in outdoor activities, school activities, and screen time, respectively, compared to 35%, 36%, and 57% of children who experienced increases in these activities. More children experienced decreases in (46% and 35%) in time spent with friends and extracurricular activities, respectively, compared to 22% and 25% of children who experienced increases in these activities. 

### 3.2. Quantitative Results 

#### 3.2.1. Physical Health 

Here, we present our results in relative risk ratios and describe them as a percent increase or decrease in the chance of an event occurring. Table 2 presents information about the associations between changes in child activities and changes in PH. For changes in PH, we found heterogeneous results with decreases in outdoor and school activities and increases in time spent with friends. Compared to children whose outdoor activity remained the same, children whose outdoor activity decreased were associated with a 163% increase in the chance of experiencing worse (RRR 2.63, *p* = 0.002) PH and a 133% increase in the chance of experiencing better PH (RRR 2.33, *p* = 0.007) relative to no change in PH. Similarly, compared to children whose school activity remained the same, children whose school activity decreased were associated with a 151% increase in the chance of experiencing worse (RRR 2.51, *p* = 0.003) PH and a 130% increase in the chance of experiencing better PH (RRR 2.30, *p* = 0.012) relative to no change in PH. Conversely, compared to children whose time with friends remained the same, children whose time with friends increased were associated with a 256% increase in the chance of experiencing worse (RRR 3.56, *p* = 0.005) PH and a 260% increase in the chance of experiencing better (RRR 3.60, *p* < 0.001) PH relative to no change in PH. Changes in screen time also showed heterogenous results. Compared to children whose screen time stayed the same, children whose screen time either decreased or increased were associated with a 156% increase in chance of experiencing worse (RRR 2.56, *p* = 0.035) PH and a 123% increase in the chance of experiencing worse PH (RRR 2.23, *p* = 0.027), respectively, relative to no change in PH. Compared to children whose activity remained the same, increases in outdoor (RRR 2.36, *p* = 0.003), school (RRR 3.07, *p* < 0.001), and extracurricular activity (RRR 3.05, *p* < 0.001) were all associated with increases in the chance of experiencing better PH relative to no change in PH. 

Table 2 also shows the relationships between demographic factors and PH. Race, spouse employment, school plan, and school quality were associated with changes in PH. We found heterogeneous results for school quality pre-COVID-19 with changes in MH. Poor school quality pre-COVID-19 compared to average school quality pre-COVID-19, was associated with a 224% increase in the chance of experiencing worse (RRR 3.24, *p* = 0.008) PH and a 188% increase in the chance of experiencing better PH (RRR 2.88, *p* = 0.024) relative to no change in PH. The only factor associated with worse PH was Hispanic ethnicity. For simplicity, we refer to children of White respondents as ‘White children’, children of Black respondents as ‘Black children’, children of Hispanic respondents as ‘Hispanic children’, and children of Asian respondents as ‘Asian children’. Compared to White children, Hispanic children were associated with an 81% increase in the chance of experiencing worse PH (RRR 1.81, *p* = 0.044) relative to no change in PH. Good school quality during COVID-19 compared to average school quality was associated with a 119% increase in the chance of better PH (RRR 2.19, *p* = 0.003). Compared to children whose school plan was in-person only, children whose school plan was online only were associated with a 116% increase in the chance of experiencing better PH (RRR 2.16, *p* = 0.015) relative to no change in PH. Compared to children who lived in families where the spouse worked full time, children living in families where the spouse was not working were associated with a 53% decrease in the chance of experiencing better PH (RRR 0.47, *p* = 0.028).

#### 3.2.2. Mental Health 

Considering the relationship between activities and changes in MH, we found heterogeneous results for changes in school activity and a decrease in screen time (Table 2). Children whose school activity decreased compared to staying the same were associated with a 410% increase in the chance of experiencing worse (5.10, *p* < 0.001) MH and a 135% increase in experiencing better (RRR 2.35, *p* = 0.018) MH, relative to no change in MH. Similarly, compared to children whose school activity remained the same, children whose school activity increased were associated with a 94% increase in the chance of experiencing worse (1.94, *p* = 0.016) MH and a 313% increase in the chance of experiencing better (RRR 4.13, *p* < 0.001) MH, relative to no change in MH. Compared to no change in screen time, a decrease in screen time was likewise associated with a 205% increase in the chance of worse (RRR 3.05, *p* = 0.007) MH and a 156% increase in the chance of better (RRR 2.56, *p* = 0.019) MH, relative to no change in MH. Compared to children whose activity remained the same, children whose outdoor activities (RRR 2.20, *p* = 0.007) and time spent with friends (RRR 3.23, *p* < 0.001) decreased were associated with an increase in the chance of worse MH, relative to no change in MH. Better MH was more likely for children whose time spent with friends (RRR 3.34, *p* < 0.001) and in extracurricular activities (RRR 4.48, *p* < 0.001) increased compared to staying the same, relative to no change in MH. 

In reviewing the relationship between demographic factors and MH, like our PH results, race/ethnicity, spouse employment, school plan, and school quality were associated with changes in MH. We found heterogenous results for children whose school plan consisted of a mix of in-person and online instruction compared to in-person only instruction. These children were associated with a 91% increase in the chance of experiencing worse (RRR 1.91, *p* = 0.043) MH and a 98% increase in the chance of experiencing better (RRR 1.98, *p* = 0.034) MH, relative to no change in MH. The only factor associated with worse MH was pre-COVID-19 school quality. Poor pre-COVID-19 school quality compared to average pre-COVID-19 school quality was associated with a 134% increase in the chance of experiencing worse (RRR 2.34, *p* = 0.044) MH, relative to no change in MH. Children had a greater chance of experiencing better MH relative to no change in MH if their school quality during COVID-19 was good, compared to average (RRR 2.11, *p* = 0.008), and if their school plan consisted of online only instruction compared to in-person instruction (RRR 2.50, *p* = 0.005). 

Race/ethnicity and spouse employment were both associated with children being less likely to experience better MH. Compared to White children, Black children were associated with a 54% decrease in the chance of experiencing better MH (RRR 0.46, *p* = 0.042) relative to no change in MH. Children living in families with the spouse not working compared to working full time were associated with a 50% decrease in the chance of experiencing better MH (RRR 0.50, *p* = 0.048) relative to no change in MH. 

#### 3.2.3. Interactions by Race/ethnicity

**Figure 1:**
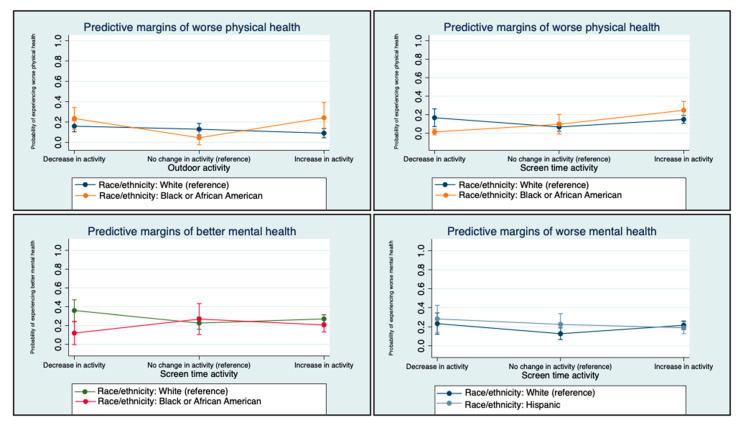
Interaction charts by race/ethnicity.

We found significant interactions by race/ethnicity for PH and MH with outdoor activity and screen time (Table 3 and Table 4). Here, we present the marginal probabilities by race/ethnicity. As seen in Figure 1, when compared to White children, Black children whose outdoor activity increased had a higher probability of experiencing worse PH relative to no change in PH (marginal probability: 0.24 for Black children compared to 0.09 for White children). Conversely, when compared to White children, Black children whose screen time decreased had a lower probability of experiencing worse PH relative to no change in PH (marginal probability: 0.01 for Black children compared to 0.18 for White children). These results suggest that increasing outdoor activity was less effective for maintaining PH for Black children compared to White children, while decreasing screen time was more effective for maintaining PH for Black children than for White children. As seen in Figure 1, When compared to White children, Black children whose screen time decreased had a lower probability of experiencing worse MH relative to no change in MH (marginal probability: 0.17 for Black children compared to 0.35 for White children). Interestingly, increased screen time was less problematic for worse MH among Hispanic children. When compared to White children, Hispanic children whose screen time increased had a lower probability of experiencing worse MH relative to no change in MH (marginal probability: 0.19 for Hispanic children compared to 0.22 for White children).

## 4. Discussion

Understanding the relationship between changes in child activities and changes in health during the pandemic is necessary as families, schools, and communities consider their responses to future waves of COVID-19 and subsequent pandemics or similar disruptions to learning. In addition to being one of the first studies to examine the relationship between changes in activities and both mental and physical health in the United States, we extend previous research that tends to view changes in activities and health as occurring in one direction (e.g., increases in activities; decreases in health) and uniformly across populations (i.e., similar results for all children). By using multinomial logistic regression and interactions across race/ethnicity, we examine *both* increases and decreases in child activities during the pandemic and better *or* worse health, as well as how these relationships are moderated by race/ethnicity. This allows us to examine heterogeneity across these constructs, ultimately capturing the complexity of these relationships during the pandemic, while highlighting implications for equity and policy. 

Across PH and MH, we had both expected and unexpected findings. While we expected a decrease in outdoor activity to be positively associated with worse PH, as well as an increase in outdoor activity to be positively associated with better PH, we were surprised to see a positive association between a decrease in outdoor activity and better PH. As a decrease in outdoor activities may signal broader COVID-19 precautions, this heterogeneity may suggest protection from potential diseases (e.g., COVID-19). Based on the previous literature linking outdoor activity and MH, our findings that a decrease in outdoor activity was positively associated with worse MH was expected [41]. Given that our findings support the previous literature that decreases in outdoor activities may relate to worse MH, interventions and policies should be implemented to promote safe outdoor activity for students. For example, state and local education agencies and schools can implement school wellness policies to promote daily outdoor breaks for students [42]. Additionally, communities should implement policies such as Safe Routes To Schools and Complete Streets, which change the built environment around schools and in neighborhoods in ways that promote outdoor play and physical activity [42,43,44,45].

When considering school activities, we were surprised to find heterogeneity in the relationships with health. As expected, an increase in school activities was associated with better PH, potentially signaling active learning or positive mind–body relationships. However, a decrease in school activities was positively associated with both worse and better PH, and both a decrease and increase in school activities was positively associated with both worse and better PH and MH. Although the relationships were larger across our expected outcomes, it was still surprising to find significant heterogeneity in these relationships. Nevertheless, this heterogeneity may reflect the heterogeneity of school experiences; for some students, less schoolwork may help them focus on other interests that improve their well-being; for others, this lack of structure may provide a sense of aimlessness. Conversely, increased schoolwork may provide a sense of purpose for some students, but for others, schoolwork may cause stress and anxiety. 

Regarding time with friends, our findings were mostly expected: a decrease in time with friends was positively associated with worse MH, and an increase in time with friends was positively associated with better MH. While there was some heterogeneity among increased time with friends and PH, this could reflect the differences in how children spend time with friends (e.g., playing physical sports vs. playing video games). While COVID-19 restrictions across the United States were being lifted during the time of this study, our findings suggest schools and parents should prioritize child and adolescent socialization even if in-person meetings may not be possible. For example, parents could promote outdoor playtime and virtual social events. 

Heterogeneity was more prevalent with screen time: both an increase and decrease in screen time were positively associated with worse PH, and a decrease in screen time was positively associated with both worse and better MH. While those that experienced increased screen time may spend less time engaging in physical activity, for others, decreased screen time may signal unobserved family hardships that ultimately relate to decreases in PH as well. At the same time, decreased screen time may signal fewer social media interactions for some (relating to worse MH) and greater in-person interactors for others (related to better MH) [46]. Given the heterogeneity of our findings, more research is needed to tease apart types of screen time and the mechanisms by which screen time may influence PH and MH. Finally, increased extracurricular activities was positively associated with better PH and MH, as expected. Schools should make extracurricular activities accessible and affordable for all students and work to create environments where students can safely engage in these activities. Increasing access to extracurriculars may not only potentially promote PH and MH but may also support outdoor activity and time spent with friends. 

When considering race/ethnicity, our findings show that Hispanic children are positively associated with worse PH during the pandemic, while Black children are negatively associated with better MH. These findings are supported by research, demonstrating the disproportionate impact of COVID-19 on minority families [19]. Additionally, we explored how race/ethnicity moderates the relationship between child activities and health. Here, we find that an increase in outdoor activities was associated with higher probabilities of having worse PH for Black children when compared to White children. This may reflect behaviors that could lead to an increased risk of COVID-19 infections or environmental conditions (e.g., poor air quality) associated with minoritized populations [47]. To address these barriers to outdoor activity, policies promoting outdoor activities should be implemented with an equity lens and rely on community-based approaches [48]. An example of this is the Our Voice citizen science model, which is a community-based participatory approach that has been implemented in marginalized communities around the world [49]. These approaches incorporate community participation along the research continuum from identifying ways to improve the environment for health to advocacy and policy change [49]. We also find significant moderation in screen time. Specifically, a decrease in screen time was associated with lower probabilities of having worse PH for Black children when compared to White children. Here, Black children who experienced a decrease in screen time may have been more likely to replace screen time with activities (e.g., outdoor) that improved PH. Conversely, a decrease in screen time was associated with lower probabilities of having better MH for Black children when compared to White children. Similarly, an increase in screen time was associated with lower probabilities of having worse MH for Hispanic children when compared to White children. Here, Black and Hispanic children may have used screen time for different purposes than White children (e.g., videos games vs. social media) [13]. These findings support the need for more research to explore how different types of screen time use relate to PH and MH, the mechanisms by which screen time influences PH and MH, and how these may differ across race/ethnicity. 

### Limitations

Despite the novel contribution of this research, we were not able to observe children directly; rather, parents reported on their perceptions of characteristics that they observe in their children. This measurement does allow for potentially biased responses. Additionally, due to sample size limitations, we were not able to speak to some of the interactions associated with Asian racial/ethnic groups, nor were we able to leverage information on “other” racial/ethnic groups. We also did not explore how other important characteristics, such as gender and age, might moderate the relationship between changes in child activities and child PH and MH. While we did control for these factors in our models, moderation by gender and age was outside the scope of this analysis. Future research in this area should explore the role of gender and age in the relationship between changes in activities and PH and MH. Finally, as there may be some unobservable characteristics that are also associated with child activities and health, we cannot establish causality with our results.

## 5. Conclusions

We explored the relationship between changes in child activities and parents’ perceptions of their child’s PH and MH during the COVID-19 pandemic and how these relationships varied by race/ethnicity. Outdoor activities, school activities, extracurricular activities, and spending time with friends were positively related to PH and MH. However, except for extracurricular activities, heterogeneous relationships were frequently observed. When compared to no change in activities, increases and decreases in activities were often simultaneously related to better and worse health. The complexity of these relationships during the pandemic were further illuminated with significant race/ethnicity interactions for PH and MH across outdoor activities and screen time. Given our findings that time spent with friends and undertaking outdoor, school, and extracurricular activities were generally positively related to PH and MH, families, neighborhood organizations, and schools should employ policies and programs that—to the greatest extent possible—safely facilitate these activities. At the same time, given the conflicting findings around screen time and outdoor activities, more research must be carried out to understand how children are using technology and engaging in outdoor activities and how the relationship between these activities and health may vary across use types and child characteristics. 

## Figures and Tables

**Table 1:** Descriptive statistics for analytic sample (n = 853) by race/ethnicity.

	(1)	(2)	(3)	(4)	(5)
	Total n = 853	White n = 491 (57.56%)	Black n = 98 (11.49%)	Hispanic n = 201 (23.56%)	Asian n= 63(7.39%)
**Outcomes, n (%)**					
Mental Health: Worse	168 (19.70)	96 (19.55)	19 (19.39)	39 (19.40)	14 (22.22)
Mental Health: No change	473 (55.45)	269 (54.79)	60 (61.22)	105 (52.24)	39 (61.90)
Mental Health: Better	212 (24.85)	126 (25.66)	19 (19.39)	57 (28.36)	10 (15.87)
Physical Health: Worse	128 (15.01)	59 (12.02)	24 (24.49)	35 (17.41)	10 (15.87)
Physical Health: No change	476 (55.80)	284 (57.84)	54 (55.10)	97 (48.26)	41 (65.08)
Physical Health: Better	249 (29.19)	148 (30.14)	20 (20.41)	69 (34.33)	12 (19.05)
**Child Activities, n (%)**					
Outdoor activity: Decrease	244 (28.60)	116 (23.63)	42 (42.86)	59 (29.35)	27 (42.86)
Outdoor activity: No change	313 (36.69)	190 (38.70)	28 (28.57)	70 (34.83)	25 (39.68)
Outdoor activity: Increase	296 (34.70)	185 (37.68)	28 (28.57)	72 (35.82)	11 (17.46)
School activity: Decrease	146 (17.12)	89 (18.13)	14 (14.29)	37 (18.41)	6 (9.52)
School activity: No change	402 (47.13)	250 (50.92)	41 (41.84)	79 (39.30)	32 (50.79)
School activity: Increase	305 (35.76)	152 (30.96)	43 (43.88)	85 (42.29)	25 (39.68)
Time with friends: Decrease	394 (46.19)	217 (44.20)	50 (51.02)	94 (46.77)	33 (52.38)
Time with friends: No change	275 (32.24)	164 (33.40)	28 (28.57)	67 (33.33)	16 (25.40)
Time with friends: Increase	184 (21.57)	110 (22.40)	20 (20.41)	40 (19.90)	14 (22.22)
Screen time: Decrease	82 (9.61)	42 (8.55)	10 (10.20)	26 (12.94)	4 (6.35)
Screen time: No change	284 (33.29)	174 (35.44)	25 (25.51)	63 (31.34)	22 (34.92)
Screen time: Increase	487 (57.09)	275 (56.01)	63 (64.29)	112 (55.72)	37 (58.73)
Extracurricular activity: Decrease	298 (34.94)	169 (34.42)	34 (34.69)	67 (33.33)	28 (44.44)
Extracurricular activity: No change	344 (40.33)	199 (40.53)	40 (40.82)	81 (40.30)	24 (38.10)
Extracurricular activity: Increase	211 (24.74)	123 (25.05)	24 (24.49)	53 (26.37)	11 (17.46)
**Covariates, n (%)**					
Gender: Female	458 (53.69)	225 (45.82)	69 (70.41)	128 (63.68)	36 (57.14)
Child grade: Pre-school	74 (8.68)	41 (8.35)	11 (11.22)	19 (9.45)	3 (4.76)
Child grade: Kindergarten	73 (8.56)	38 (7.74)	11 (11.22)	18 (8.96)	6 (9.52)
Child grade: 1st through 2nd grade	77 (9.03)	40 (8.15)	11 (11.22)	19 (9.45)	7 (11.11)
Child grade: 3rd through 5th grade	176 (20.63)	106 (21.59)	15 (15.31)	44 (21.89)	11 (17.46)
Child grade: 6th through 8th grade	168 (19.70)	198 (19.96)	20 (20.41)	39 (19.40)	11 (17.46)
Child grade: 9th through 12th grade	285 (33.41)	168 (34.22)	30 (30.61)	62 (30.85)	25 (39.68)
Income: (0–50%) AMI	219 (25.67)	108 (22.00)	37 (37.76)	58 (28.86)	16 (25.40)
Income: (50–80%) AMI	148 (17.35)	85 (17.31)	16 (16.33)	40 (19.90)	7 (11.11)
Income: (80–120%) AMI	194 (22.74)	107 (21.79)	22 (22.45)	46 (22.89)	19 (30.16)
Income: (120%+) AMI	292 (34.23)	191 (38.90)	23 (23.47)	57 (28.36)	21 (33.33)
Job/income loss: Yes	293 (34.35)	156 (31.77)	31 (31.63)	90 (44.78)	16 (25.40)
Bachelor’s degree: Yes	662 (77.61)	380 (77.39)	79 (80.61)	146 (72.64)	57 (90.48)
Primary language: English	814 (95.43)	489 (99.59)	96 (97.96)	174 (86.57)	55 (87.30)
Primary language: Spanish/Other	39 (4.57)	2 (0.41)	2 (2.04)	27 (13.43)	8 (12.70)
Children: One child	395 (46.31)	222 (45.21)	54 (55.10)	85 (42.29)	34 (53.97)
Children: More than one child	458 (53.69)	269 (54.79)	44 (44.90)	116 (57.71)	29 (46.03)
Spouse employment: Single	155 (18.17)	70 (14.26)	37 (37.76)	39 (19.40)	9 (14.29)
Spouse employment: Working full time	492 (57.68)	291 (59.27)	43 (43.88)	121 (60.20)	37 (58.73)
Spouse employment: Working part time	86 (10.08)	52 (10.59)	7 (7.14)	19 (9.45)	8 (12.70)
Spouse employment: Not working	120 (14.07)	78 (15.89)	11 (11.22)	22 (10.95)	9 (14.29)
Employment: Working full time	602 (70.57)	356 (72.51)	68 (69.39)	138 (68.66)	40 (63.49)
Employment: Working part time	102 (11.96)	52 (10.59)	10 (10.20)	32 (15.92)	8 (12.70)
Employment: Not working	149 (17.47)	83 (16.90)	20 (20.41)	31 (15.42)	15 (23.81)
School plan: In-person only	189 (22.16)	132 (26.88)	13 (13.27)	37 (18.41)	7 (11.11)
School plan: Online only	262 (30.72)	127 (25.87)	44 (44.90)	69 (34.33)	22 (34.92)
School plan: Mix of in-person and online	314 (36.81)	182 (37.07)	31 (31.63)	76 (37.81)	25 (39.68)
School plan: Choice of in-person and online	88 (10.32)	50 (10.18)	10 (10.20)	19 (9.45)	9 (14.29)
School quality pre COVID: Poor	61 (7.15)	36 (7.33)	13 (13.27)	10 (4.98)	2 (3.17)
School quality pre COVID: Average	230 (26.96)	114 (23.22)	30 (30.61)	64 (31.84)	22 (34.92)
School quality pre COVID: Good	562 (65.89)	341 (69.45)	55 (56.12)	127 (63.18)	39 (61.90)
School quality during COVID: Poor	87 (10.20)	56 (11.41)	9 (9.18)	17 (8.46)	5 (7.94)
School quality during COVID: Average	261 (30.60)	125 (25.46)	39 (39.80)	74 (36.82)	23 (36.51)
School quality during COVID: Good	505 (59.20)	310 (63.14)	50 (51.02)	110 (54.73)	35 (55.56)
Online learning tool at home: Yes	548 (64.24)	318 (64.77)	57 (58.16)	131 (65.17)	42 (66.67)
Adult learning assistance at home: Yes	404 (47.36)	247 (50.31)	47 (47.96)	85 (42.29)	25 (39.68)
Broadband at home: Yes	579 (67.88)	341 (69.45)	66 (67.35)	131 (65.17)	41 (65.08)
Quiet place to study at home: Yes	533 (62.49)	308 (62.73)	65 (66.33)	119 (59.20)	41 (65.08)
Cumulative COVID-19 incidences (County),^a^ M (SD)	10.90 (1.61)	10.61 (1.62)	11.06 (1.64)	11.43 (1.46)	11.28 (1.50)
Population Density (Thousands per Sq. Mile), M (SD)	1.55 (2.15)	1.42 (2.12)	1.76 (2.19)	1.49 (1.57)	2.51 (3.50)

^a^ Cumulative number of cases per county, logged.

**Table 2:** Association of changes in child and adolescent activities with changes in physical and mental health during COVID-19.

	(1)				(2)			
	Physical Health: Worse		Physical Health: Better		Mental Health: Worse		Mental Health: Better	
	RRR	(se)	RRR	(se)	RRR	(se)	RRR	(se)
Outdoor activity: Decrease	2.63 **	0.83	2.33 **	0.73	2.20 **	0.64	1.69	0.56
Outdoor activity: Increase	1.36	0.46	2.36 **	0.67	1.45	0.44	1.64	0.51
School activity: Decrease	2.51 **	0.77	2.30 *	0.76	5.10 ***	1.47	2.35 *	0.85
School activity: Increase	1.34	0.40	3.07 ***	0.81	1.94 *	0.53	4.13 ***	1.14
Time with friends: Decrease	2.01	0.74	0.67	0.20	3.23 ***	1.07	1.01	0.33
Time with friends: Increase	3.56 **	1.60	3.60 ***	1.17	2.19	0.92	3.34 ***	1.09
Screen time: Decrease	2.56 *	1.14	1.88	0.71	3.05 **	1.27	2.56 *	1.02
Screen time: Increase	2.23 *	0.81	1.18	0.34	1.56	0.50	1.28	0.39
Extracurricular activity: Decrease	1.05	0.30	0.69	0.20	0.95	0.25	1.18	0.38
Extracurricular activity: Increase	0.55	0.23	3.05 ***	0.88	0.89	0.32	4.48 ***	1.35
Race/ethnicity: Black	1.42	0.51	0.56	0.21	0.67	0.24	0.46 *	0.18
Race/ethnicity: Asian	1.12	0.52	0.58	0.27	1.09	0.46	0.39	0.19
Race/ethnicity: Hispanic	1.81 *	0.54	1.4	0.38	1.07	0.30	0.87	0.25
Gender: Female	0.94	0.25	0.73	0.17	1.01	0.25	0.76	0.19
Child grade: Kindergarten	0.82	0.48	0.66	0.34	0.50	0.30	1.08	0.55
Child grade: 1st through 2nd grade	0.55	0.33	0.86	0.43	0.95	0.53	1.65	0.83
Child grade: 3rd through 5th grade	0.78	0.39	1.06	0.46	0.89	0.43	0.85	0.37
Child grade: 6th through 8th grade	0.73	0.37	0.89	0.39	1.05	0.50	1.17	0.52
Child grade: 9th through 12th grade	0.9	0.43	1.06	0.44	1.56	0.71	0.82	0.35
Income: (50–80%) AMI	0.75	0.26	0.91	0.3	1.50	0.49	0.77	0.26
Income: (80–120%) AMI	0.58	0.21	1.10	0.36	0.95	0.33	0.68	0.23
Income: (120%+) AMI	0.84	0.29	0.92	0.30	1.33	0.45	0.56	0.19
Job/income loss: Yes	1.56	0.39	1.54	0.35	1.21	0.29	1.11	0.27
Bachelor’s degree: Yes	0.56	0.18	0.77	0.23	0.67	0.20	1.18	0.38
Primary language: Spanish/Other	0.36	0.21	0.49	0.28	0.57	0.32	2.05	1.07
Children: More than one child	0.88	0.21	1.33	0.29	0.83	0.18	1.31	0.30
Spouse employment: Working part time	1.23	0.50	1.73	0.60	1.69	0.63	1.79	0.67
Spouse employment: Not working	0.64	0.24	0.47 *	0.16	0.73	0.24	0.50 *	0.18
Spouse employment: Single	1.50	0.51	0.65	0.24	0.90	0.30	0.59	0.23
Employment: Working part time	0.50	0.19	0.84	0.29	1.36	0.47	1.14	0.41
Employment: Not working	0.90	0.29	1.14	0.39	1.69	0.50	1.35	0.49
School plan: Online only	0.93	0.34	2.16 *	0.68	1.16	0.40	2.50 **	0.81
School plan: Mix of in-person and online	1.44	0.49	1.77	0.55	1.91 *	0.61	1.98 *	0.64
School plan: Choice of in-person and online	0.97	0.44	2.04	0.88	1.29	0.54	1.50	0.72
School quality pre-COVID: Poor	3.24 **	1.44	2.88 *	1.35	2.34 *	0.99	0.88	0.44
School quality pre-COVID: Good	0.87	0.25	1.36	0.40	0.84	0.24	1.00	0.31
School quality during COVID: Poor	1.69	0.59	1.10	0.47	1.34	0.47	1.57	0.71
School quality during COVID: Good	0.91	0.26	2.19 **	0.59	0.76	0.20	2.11 **	0.60
Cumulative COVID-19 incidences (County) ^a^	1.14	0.09	1.12	0.08	0.98	0.07	1.07	0.08
Population Density (Thousands per Sq. Mile)	0.99	0.06	0.99	0.05	0.96	0.05	0.95	0.05
Online learning tool at home: Yes	0.94	0.24	1.04	0.25	0.69	0.16	0.86	0.22
Adult learning assistance at home: Yes	0.91	0.23	0.69	0.17	0.95	0.23	0.72	0.19
Broadband at home: Yes	1.21	0.33	1.32	0.33	1.1	0.28	0.96	0.24
Quiet place to study at home: Yes	0.85	0.23	0.95	0.22	1.43	0.36	1.46	0.36
Observations	853				853			
Pseudo *R*^2^	0.32				0.32			

^a^ Cumulative number of cases per county, logged; Relative Risk Ratios (RRR) provided; Standard errors in parentheses; Reference categories: Outdoor activity (No change); School activity (No change); Time with friends (No change); Screen time (No change); Extracurricular activity (No change); Race/ethnicity (White); Gender (Male); Child grade (Pre-school); Income (0–50% AMI); Bachelor’s degree (No); Primary language (English); Married/living with partner (No); Children (One child); Spouse employment (Working full time); Employment (Working full time); School Plan (In-person only); School quality pre-COVID (Average); School quality during COVID (Average); Online learning tool at home (No);Adult learning assistance at home (No); Broadband at home (No); Quiet place to study at home (No); * *p* < 0.05, ** *p* < 0.01, *** *p* < 0.001.

**Table 3:** Association of changes in child and adolescent activities with changes in physical health during COVID-19 with interactions by race/ethnicity.

	(1)				(2)				(3)				(4)				(5)			
	Outdoor Activity × Race/ethnicity				School Activity × Race/ethnicity				Time with Friends × Race/ethnicity				Screen Time × Race/ethnicity				Extracurricular Activity × Race/ethnicity			
	Physical Health				Physical Health				Physical Health				Physical Health				Physical Health			
	Worse		Better		Worse		Better		Worse		Better		Worse		Better		Worse		Better	
	RRR	(se)	RRR	(se)	RRR	(se)	RRR	(se)	RRR	(se)	RRR	(se)	RRR	(se)	RRR	(se)	RRR	(se)	RRR	(se)
Race/ethnicity: Black	0.23	0.23	0.46	0.35	1.44	0.79	0.81	0.49	1.66	1.33	0.58	0.39	1.63	1.41	1.10	0.77	0.70	0.43	0.63	0.35
Race/ethnicity: Asian	-	-	0.06	0.44	1.00	0.69	0.45	0.38	1.71	1.97	0.95	0.81	0.77	0.94	0.13*	0.13	0.72	0.64	0.32	0.24
Race/ethnicity: Hispanic	1.46	0.75	1.65	0.76	1.23	0.57	1.09	0.48	1.27	0.81	1.05	0.46	3.16	1.97	1.00	0.48	1.72	0.80	1.08	0.46
Outdoor activity: Decrease	1.61	0.67	2.02	0.86	2.82 **	0.90	2.38 **	0.75	2.47 **	0.79	2.31 **	0.74	2.29 *	0.74	2.13 *	0.69	2.66 **	0.85	2.41 **	0.77
Outdoor activity: Increase	0.81	0.37	2.75 **	1.02	1.40	0.48	2.37 **	0.68	1.30	0.45	2.35 **	0.67	1.21	0.42	2.31 **	0.67	1.41	0.49	2.33 **	0.67
School activity: Decrease	2.67 **	0.84	2.38 **	0.79	2.06	0.81	2.13	0.85	2.49 **	0.77	2.32 *	0.77	2.38 **	0.74	2.32 *	0.78	2.53 **	0.78	2.23*	0.74
School activity: Increase	1.47	0.45	3.07 ***	0.82	1.17	0.53	2.90 **	0.99	1.31	0.39	3.16 ***	0.85	1.31	0.39	3.03 ***	0.81	1.35	0.40	3.18 ***	0.85
Time with friends: Decrease	2.07 *	0.77	0.66	0.20	2.01	0.74	0.65	0.20	1.79	0.85	0.59	0.21	2.34 *	0.87	0.69	0.21	2.13 *	0.78	0.66	0.20
Time with friends: Increase	3.48 **	1.63	3.50 ***	1.15	3.55 **	1.61	3.55 ***	1.16	4.12 *	2.55	3.67 **	1.53	4.63 ***	2.14	4.29 ***	1.43	3.49 **	1.61	3.77 ***	1.24
Screen time: Decrease	3.04 *	1.40	1.92	0.73	2.45*	1.11	1.90	0.73	3.07 *	1.40	1.93	0.73	4.44 *	2.89	2.30	1.18	2.71*	1.22	1.95	0.74
Screen time: Increase	2.36 *	0.87	1.19	0.35	2.18 *	0.80	1.17	0.35	2.48 *	0.92	1.19	0.35	2.85 *	1.43	0.94	0.33	2.26*	0.82	1.2	0.35
Extracurricular activity: Decrease	1.10	0.32	0.69	0.21	1.02	0.30	0.68	0.20	1.05	0.30	0.68	0.20	1.01	0.29	0.67	0.20	0.80	0.31	0.67	−0.24
Extracurricular activity: Increase	0.54	0.23	3.14 ***	0.91	0.56	0.24	3.11 ***	0.90	0.57	0.24	3.05 ***	0.88	0.49	0.21	3.15 ***	0.92	0.52	0.31	2.24 *	0.84
Activity: Decrease × Black	7.55	8.28	1.89	1.86	0.65	0.58	0.25	0.34	1.20	1.07	0.96	0.85	0.02 *	0.03	0.11	0.13	3.27	2.47	0.27	0.33
Activity: Decrease × Asian	-	-	-	-	2.33	2.74	-	-	0.47	0.61	0.59	0.64	2.08	4.03	4.40	7.54	2.18	2.27	0.68	0.91
Activity: Decrease × Hispanic	1.71	1.14	1.44	0.94	2.42	1.71	2.16	1.53	1.64	1.18	1.65	0.93	0.69	0.66	1.09	0.89	1.00	0.59	1.33	0.78
Activity: Increase × Black	14.68 *	17.18	1.11	1.06	1.12	0.88	0.69	0.53	0.15	0.20	0.63	0.59	1.21	1.13	0.53	0.43	1.54	1.78	1.23	1.01
Activity: Increase × Asian	-	-	-	-	0.84	0.88	1.73	1.77	1.50	2.25	0.48	0.56	1.68	2.24	8.43	9.90	-	-	6.70	7.61
Activity: Increase × Hispanic	1.02	0.75	0.51	0.31	1.61	1.09	1.31	0.75	1.38	1.46	1.36	1.05	0.47	0.33	1.87	1.08	1.11	1.03	1.76	1.14
Model Covariates Included	Y				Y				Y				Y				Y			
Observations	853				853				853				853				853			
Pseudo *R*^2^	0.33				0.32				0.32				0.33				0.33			

Notes: Relative Risk Ratios (RRR) provided; Standard errors in parentheses; Coefficients for interactions of Asian and outdoor activity were deviant and thus were not displayed due to small sample size of Asians in these cells. All other model covariates, as listed in Table 2, are included in the above interaction models. * *p* < 0.05, ** *p* < 0.01, *** *p* < 0.001.

**Table 4:** Association of changes in child and adolescent activities with changes in mental health during COVID-19 with interactions by race**/ethnicity**.

	(1)				(2)				(3)				(4)				(5)			
	Outdoor activity × Race/ethnicity				School activity × Race/ethnicity				Time with friends × Race/ethnicity				Screen time × Race/ethnicity				Extracurricular activity × Race/ethnicity			
	Mental Health				Mental Health				Mental Health				Mental Health				Mental Health			
	Worse		Better		Worse		Better		Worse		Better		Worse		Better		Worse		Better	
	RRR	(se)	RRR	(se)	RRR	(se)	RRR	(se)	RRR	(se)	RRR	(se)	RRR	(se)	RRR	(se)	RRR	(se)	RRR	(se)
Race/ethnicity: Black	0.85	0.65	1.04	0.75	1.10	0.59	0.32	0.28	2.21	1.69	0.68	0.51	2.02	1.63	1.70	1.34	1.42	0.76	0.22	0.18
Race/ethnicity: Asian	1.50	1.15	0.85	0.62	0.96	0.59	0.19	0.21	1.82	2.08	0.43	0.50	2.08	1.86	0.53	0.49	2.15	1.54	0.24	0.21
Race/ethnicity: Hispanic	1.60	0.77	1.23	0.66	0.77	0.35	0.99	0.48	1.90	1.08	0.58	0.30	2.74	1.52	1.60	0.84	1.39	0.63	0.61	0.3
Outdoor activity: Decrease	3.55 **	1.38	2.84 *	1.29	2.31 **	0.68	1.68	0.55	2.40 **	0.71	1.75	0.58	2.53 **	0.76	1.65	0.56	2.18 **	0.64	1.66	0.55
Outdoor activity: Increase	1.36	0.54	2.13	0.87	1.45	0.45	1.64	0.51	1.43	0.44	1.61	0.50	1.53	0.48	1.55	0.49	1.36	0.42	1.59	0.50
School activity: Decrease	4.92 ***	1.43	2.34 *	0.85	4.12 ***	1.48	2.20	0.98	5.15 ***	1.50	2.32 *	0.85	4.79 ***	1.40	2.24 *	0.82	5.32 ***	1.56	2.26*	0.83
School activity: Increase	1.99 *	0.55	4.17 ***	1.15	2.43 *	0.93	4.13 ***	1.45	2.05 **	0.57	4.35 ***	1.22	1.96 *	0.54	4.25 ***	1.18	2.02 *	0.55	4.19 ***	1.17
Time with friends: Decrease	3.37 ***	1.13	1.03	0.34	3.33 ***	1.11	1.00	0.33	5.18 ***	2.25	1.03	0.41	3.31 ***	1.13	1.06	0.35	3.14 ***	1.05	1.01	0.33
Time with friends: Increase	2.03	0.87	3.27 ***	1.07	2.11	0.89	3.24 ***	1.06	2.35	1.37	2.49 *	1.03	2.30	0.99	3.58 ***	1.19	2.25	0.96	3.45 ***	1.14
Screen time: Decrease	2.95 *	1.25	2.41 *	0.96	3.13 **	1.33	2.64 *	1.06	3.10 **	1.31	2.61 *	1.05	3.97 *	2.37	4.24 *	2.39	2.95 **	1.24	2.69*	1.09
Screen time: Increase	1.63	0.53	1.31	0.40	1.56	0.51	1.29	0.39	1.60	0.52	1.29	0.39	2.66*	1.09	1.86	0.71	1.56	0.50	1.34	0.41
Extracurricular activity: Decrease	0.94	0.25	1.14	0.37	0.90	0.24	1.16	0.37	0.91	0.24	1.16	0.37	0.96	0.26	1.16	0.37	1.38	0.48	0.99	0.39
Extracurricular activity: Increase	0.89	0.32	4.41 ***	1.34	0.84	0.31	4.39 ***	1.33	0.89	0.33	4.63 ***	1.40	0.84	0.31	4.49 ***	1.37	1.18	0.58	3.26 **	1.26
Activity: Decrease × Black	0.47	0.42	0.30	0.28	0.68	0.60	1.39	1.89	0.20	0.18	0.48	0.45	0.14	0.19	0.04 *	0.05	0.36	0.26	2.08	2.23
Activity: Decrease × Asian	0.37	0.35	0.29	0.33	0.75	0.88	-	-	0.36	0.46	0.67	0.92	-	-	-	-	0.44	0.39	0.58	0.86
Activity: Decrease × Hispanic	0.33	0.21	0.43	0.31	3.27	2.26	1.25	0.98	0.43	0.28	1.33	0.85	0.50	0.45	0.56	0.48	0.51	0.29	1.65	1.05
Activity: Increase × Black	1.37	1.38	0.37	0.34	0.33	0.25	1.47	1.44	0.36	0.42	0.82	0.82	0.26	0.23	0.28	0.25	-	-	2.72	2.73
Activity: Increase × Asian	2.31	2.83	0.23	0.28	1.23	1.08	3.10	3.92	2.03	2.92	1.33	1.86	0.29	0.29	0.62	0.68	-	-	3.19	3.74
Activity: Increase × Hispanic	1.00	0.66	0.83	0.55	0.91	0.58	0.75	0.45	0.83	0.84	2.85	2.18	0.26 *	0.17	0.40	0.25	1.58	1.25	1.82	1.24
Model Covariates Included	Y				Y				Y				Y				Y			
Observations	853				853				853				853				853			
Pseudo *R*^2^	0.33				0.33				0.33				0.33				0.33			

Notes: Relative Risk Ratios (RRR) provided; Standard errors in parentheses; Coefficients for interactions of Asian and outdoor activity were deviant and thus were not displayed due to small sample size of Asians in these cells. All other model covariates, as listed in Table 1, are included in the above interaction models. * *p* < 0.05, ** *p* < 0.01, *** *p* < 0.001.

## Data Availability

Given the sensitive nature of our survey, we cannot publicly share our data. However, more details can be provided upon request.
